# Insulin-like Growth Factor 1 in relation to future hearing impairment: findings from the English Longitudinal Study of Ageing

**DOI:** 10.1038/s41598-017-04526-7

**Published:** 2017-06-23

**Authors:** Camille Lassale, G. David Batty, Andrew Steptoe, Paola Zaninotto

**Affiliations:** Department of Epidemiology & Public Health, University College London, London, WC1E 6BT United Kingdom

## Abstract

Insulin-like Growth Factor 1 (IGF-1) is associated with cardiovascular disease, itself a risk factor for hearing impairment, and, in animal studies, molecular evidence suggests a role for IGF-1 in hearing function. However, the link between IGF-1 and the occurrence of hearing impairment is untested in population-based studies of humans. A total of 4390 participants aged ≥50 y (mean [SD] age 64.2 [8.0] years at baseline, 55% women) from the English Longitudinal Study of Ageing provided serum levels of IGF-1 in 2008 and again in 2012. Hearing acuity was assessed by an objective hearing test (HearCheck handheld device) in 2014 when the prevalence was 38.2%. In the full cohort, IGF-1 was not associated with subsequent hearing impairment (OR_5nmol/L increase_; 95% CI: 1.01; 0.94, 1.09). However, this relationship appeared to differ by age (p-value for interaction = 0.03). Thus, in younger participants (aged 50–60 y, n = 1400), IGF-1 was associated with lower odds of hearing impairment (0.86; 0.73, 1.00) after adjustment for a range of potential confounders. Among people ≥60 y (n = 2990) there was a non-significant ‘J’-shaped association. Our observational evidence that higher levels of IGF-1 appeared to confer some protection against hearing impairment in some older adults warrants replication in other prospective cohort studies.

## Introduction

Age-related hearing loss (presbycusis) is the most common cause of adult auditory deficiency and it is a major cause of disability^1, 2^. For example, in the United States^3^ and in the United Kingdom^4^, around two-thirds of people older than 70 years report hearing loss. As well as being an important disease entity in its own right, it has marked economic, social, and health ramifications. Thus, poor hearing has a deleterious impact on physical functioning^5^, cognitive functioning^6^, social and family life^7^ and mental health^8^. In the US, aggregate societal costs, including not only medical and assistive device costs but also reduced work productivity, were estimated to be $4.6 billion over the lifetime of affected people^9^. With the condition essentially incurable, the focus becomes identification of potentially modifiable risk factors.

Evidence from a range of scientific disciplines implicates Insulin-like Growth Factor 1 (IGF-1) in the aetiology of hearing loss. First, atherosclerosis^10, 11^ and selected cardiovascular disease risk factors – smoking^12^, diabetes^13^, overweight^12, 14^ – have been associated with hearing impairment both in cross-sectional^11, 13, 14^ and longitudinal studies^10, 12^. Lower levels of IGF-1 have been shown to be associated with elevated rates of cardiovascular disease^15^. Second, molecular data from animal studies suggest a role for IGF-1 in the development and protection of hearing function^16, 17^. Rescue of hair cells from apoptosis via IGF-1 receptor-mediated actions or by downregulation of pro-apoptotic gene expression, and regulation of glucose transporters in the outer cells have been proposed as underlying mechanisms^18^. Third, we have recently shown that physical stature, which is related to IGF-1 levels^19, 20^, is related to hearing impairment^21^, with taller individuals less likely to experience hearing loss, which is consistent with results of two other studies^22, 23^. While lower height in itself is of course not a risk factor for hearing impairment, the pre-adult characteristics that it proxies - inadequate nutrition, illness and poverty – may be.

Despite a strong *prima facie* case for a link between circulating levels of IGF-1 and hearing impairment, to our knowledge, this relationship has not been examined in a well-characterised, free-living population of humans. This was the purpose of the present study in which we utilised data on repeat measurement of IGF-1 levels and subsequent objectively measured hearing impairment in the English Longitudinal Study of Ageing (ELSA)^24^.

## Methods

### Study population

ELSA is an on-going, prospective cohort study of a representative panel of men and women living in England aged ≥50 years^24^ at recruitment in 2002/3 (wave 1). Data are collected biennially using computer-assisted personal interviews (CAPI) and self-completion questionnaires, with additional nurse visits every 4 years for the assessment of biomedical data. Ethical approval for all data collection was granted from NHS Research Ethics Committees under the National Research and Ethics Service^25^ and all participants provided informed consent. All methods were performed in accordance with approved guidelines and regulations.

**Figure 1:**
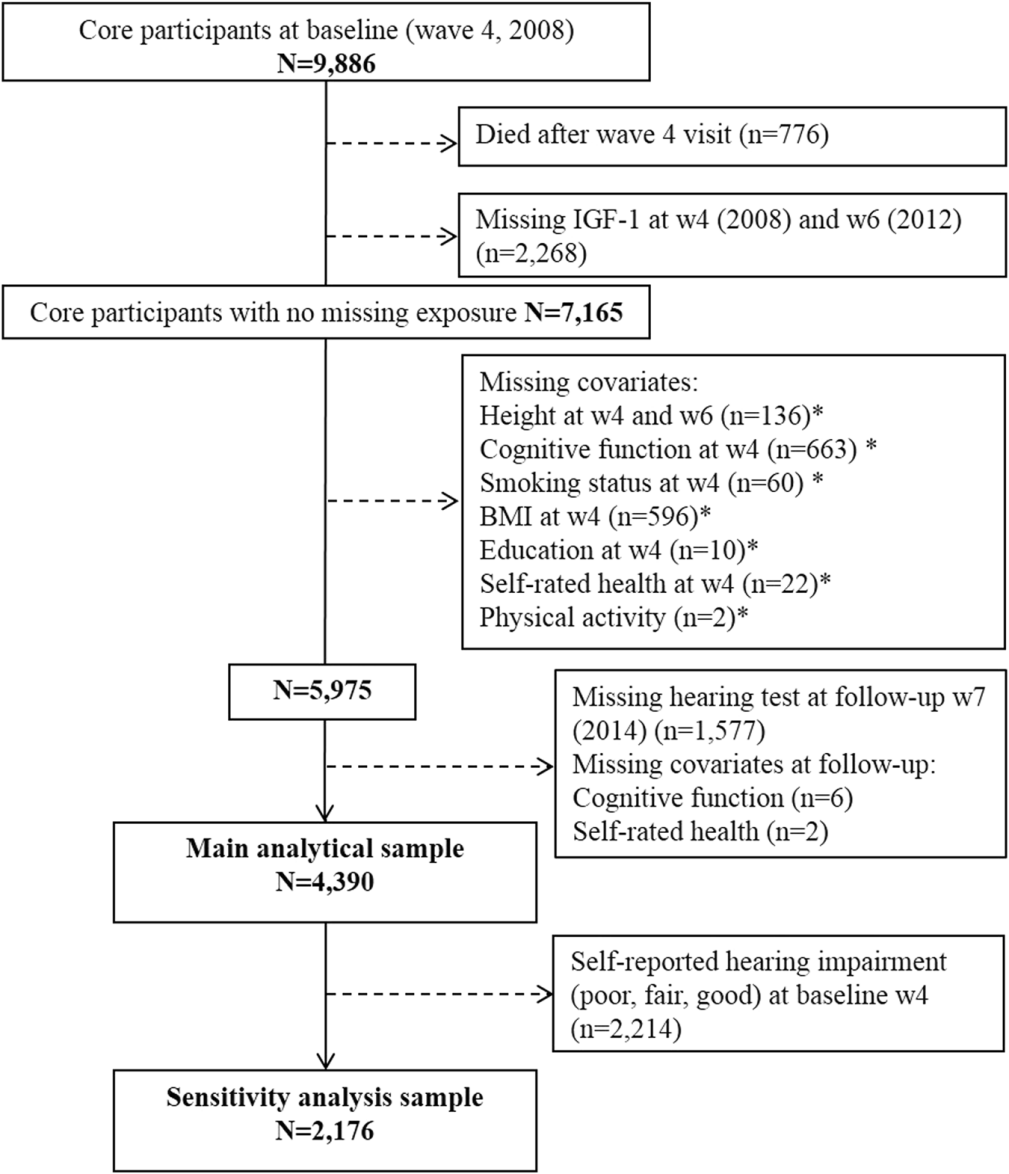
Flow of study members into the analytical sample: the English Longitudinal Study of Ageing. *Non-mutually exclusive numbers.

The ‘baseline’ of the present study was Wave 4 (2008). The analysis was based on the sample with complete data both at baseline (2008) and at follow-up (2014). As indicated in Fig. 1, of the 9886 participants attending baseline visit, 5975 had complete data on IGF-1 levels and covariates at baseline. Of them, 1577 did not have a hearing test at follow-up (wave 7, 2014), resulting in a final analytical sample of 4390 study members (44% of initial sample).

### IGF-1

Serum total IGF-1 was measured for the first time in ELSA in 2008 (Wave 4) - which represents study ‘baseline’ herewith - and 2012 (Wave 6) when blood samples were drawn from participants at rest who had not eaten, smoked, or drunk alcohol in the preceding 30 minutes. IGF-1 was batch-assayed with the DPC Immulite 2000 method^26^. Circulating IGF-1 values were reported as whole numbers (ranging from 3 to 200) expressed as nanomoles per litre (nmol/L). Total IGF-1 includes IGF-1 that is bound to IGF binding proteins (99%) and free IGF-1.

### Hearing acuity

At wave 7 (2014), all study members were invited to take part in an objective hearing test unless they reported use of a cochlear implant or had an ear infection. Hearing acuity was tested with the Siemens HearCheck^﻿™﻿^ device^27^. This screening tool has shown good sensitivity (ranging from 78% to 92%) and acceptable to good specificity (ranging from 62 to 95%) in two evaluation studies comparing results of the screener to pure tone audiometry^27, 28^. This is a simple, low-cost, handheld appliance which produces a fixed series of three pure high frequency (3 kHz) tones and three mid-frequency (1 kHz) tones. Participants who wore hearing aids were asked to remove them for the test. With the interviewer holding the HearCheck™ device against the participant’s left ear, they were asked to indicate when they heard a tone when a 1 kHz frequency sound was made at three decreasing intensities (55, 35 and 20 dB HL). This was followed by three tones (at 75, 55 and 35 dB HL) for a 3 kHz sound. Both tests were then repeated for the right ear. The main outcome, hearing impairment, was defined as hearing fewer than 6 tones in the best hearing ear, i.e. a hearing threshold of 20 dB HL at 1 kHz or 35 dB HL at 3k Hz. A secondary outcome considered in sensitivity analysis was “moderate to severe” hearing impairment, defined as hearing threshold of 35 dB HL at 1 kHz or 55 dB HL at 3k Hz. Data on use of hearing aid was recorded as ‘Yes, most of the time’, ‘Yes, sometimes’, and ‘No’. The vast majority of participants did not wear a hearing aid (n = 3767, 86%), while 8% wore a hearing aid most of the time and 6% some of the time.

Participants underwent a face-to-face CAPI at baseline (2008), including a question to self-rate the participant’s hearing (including hearing aid if wears one) on a 5-point scale (‘poor’ to ‘excellent’). Self-reported hearing impairment at baseline was defined as rating hearing as ‘poor’ or ‘fair’.

### Covariates

Covariates were also assessed through CAPI at baseline (wave 4). Self-reported health behaviours considered as covariates in the present analyses included smoking status (current, former, never) and leisure-time physical activity (5 levels from low to high). Cognitive function was assessed using a battery of tests: memory measured using a word-list learning test (immediate and delayed), and executive functioning ascertained using a word-finding task (how many different animals the participant can name in 1 minute). A summary score of memory functioning was used as covariate for cognitive function. General health was self-rated on a 5-point scale ranging from ‘poor’ to ‘excellent’. To account for change in cognitive function and in general health between baseline and assessment of hearing, we also created a change variable equal to the difference between the score at baseline and at follow-up. Depression symptoms were ascertained during the CAPI using the eight-item Center for Epidemiologic Studies Depressive CES-D scale and depression was defined as anyone scoring 4 or above^29^. Educational attainment was classified as low (compulsory schooling), medium (up to high school diploma) and high (university degree or higher). Height and weight were measured directly during the nurse visit (concomitant with the blood test) and body mass index (BMI) was defined as weight (kg) divided by height squared (m^2^).

### Statistical analysis

We used the average of the two IGF-1 measurements at both wave 4 and 6 when available, or either of these if only one was available: 747 were based only on wave 4 measurement, and 558 only on wave 6 measurements, hence 3085 (70%) were the average of wave 4 and wave 6 measurements. Taking the average of two measurements can limit fluctuations due to measurement error or biologic variability and lead to more precise estimates of the relationship with the outcome^30^, as reproducibility of IGF-1 over 4 months to 15 years can be only moderate leading to attenuation of the association if based on a single measurement^31–33^. Baseline characteristics were compared across IGF-1 quintiles (sex-specific) by analysis of covariance. To summarise the relation between IGF-1 levels and hearing impairment, we computed odds ratios (ORs) and 95% confidence intervals using logistic regression analyses. In Model 0, we adjusted the association for baseline age (continuous) and sex. In Model 1, we further adjusted the ORs for height, smoking status (3 categories), BMI (continuous), cognitive function (continuous), educational level (low, medium, high), physical activity (5 levels) and self-rated health (5 levels) – all statistically significant predictors of hearing impairment. In the main analysis, we used self-reported hearing impairment at baseline as a covariate in Model 2 (plus all factors included in Model 1), to account for pre-existing hearing impairment. In a secondary analysis, we excluded participants who self-reported hearing impairment at baseline (rated their hearing acuity as good, fair, poor), focusing on a sample likely free of hearing impairment at baseline (hearing acuity rated as very good or excellent) to model the ‘incidence’ of new cases of hearing impairment. We performed three additional sensitivity analyses using 1) the average of two IGF-1 measurements only (excluding participants with only one measurement); 2) IGF-1 measured at baseline (2008) only; 3) modelling moderate to severe hearing impairment.

To test for a linear trend, we also modelled IGF-1 as a continuous variable. We examined potential nonlinear relations using restricted cubic spline transformations^34^ and tested nonlinearity by using the likelihood ratio test, comparing nested models with a linear or linear and cubic spline terms. Computations were made using the SAS macro %lgtphcurv9^35^ for the logistic model and the p-value for curvature was reported (a p-value < 0.05 indicates non-linearity).

We explored the association of IGF-1 levels with hearing impairment according to the age at IGF-1 measurement and gender. With age-related hearing impairment being a condition whereby the majority of people are first affected around the ages of 60^36^, we used this as our threshold. All statistical analyses were performed using SAS software version 9.3 (SAS Institute Inc, Cary, NC, USA).

## Results

At baseline, the mean (SD) age of the population was 64.2 (8.0) years, ranging 50 y to 90 y, and comprised 55% women. Compared to the participants who were included in the analytical sample, people who were not included were older (mean age 68.0 [11.1] years, t-test for difference p < 0.0001), but the sex ratio was similar.

The average serum IGF-1 value was somewhat higher in men (17.1 [5.2] nmol/L than women (15.4 [5.0] nmol/L). Baseline characteristics across quintiles of IGF-1 are presented in Table 1. People with higher levels of IGF-1 were younger, taller, had a lower BMI, were more physically active, less likely to belong to the lowest wealth group and to have a basic education, and had slightly higher cognitive function (memory). A lower prevalence of diabetes, poor self-rated health and depressive symptoms was apparent in the intermediate quintiles compared to low and high quintiles.

**Table 1:** Baseline characteristics ^a^by sex-specific quintiles of serum Insulin-Like Growth Factor 1 (IGF-1) (2008 and/or 2012), the English Longitudinal Study of Ageing (n = 4390).

	Sex-specific IGF-1 Quintile^b^					P value for difference	
	1	2	3	4	5	Linear	Quadratic
Women %	56.4 (1.7)	54.3 (1.7)	52.5 (1.8)	57.7 (1.6)	51.6 (1.7)	0.25	0.89
IGF-1, nmol/L, mean	9.7 (0.1)	13.3 (0.1)	15.5 (0.1)	18.1 (0.1)	23.8 (0.1)	<0.0001	<0.0001
Age, years, mean	66.7 (0.3)	64.7 (0.3)	63.8 (0.3)	63.4 (0.3)	62.3 (0.3)	<0.0001	0.02
Height, cm, mean	164.5 (0.3)	166.0 (0.3)	166.7 (0.3)	166.4 (0.3)	167.2 (0.3)	<0.0001	0.04
BMI, kg/m^2^, mean	28.8 (0.2)	28.2 (0.2)	28.1 (0.2)	27.9 (0.2)	27.9 (0.2)	<0.0001	0.08
Diabetes, %	10.7 (0.9)	6.8 (0.9)	6.5 (0.9)	6.2 (0.9)	8.6 (0.9)	0.11	0.0002
Cardiovascular disease, %	8.4 (0.9)	8.9 (0.9)	6.8 (0.9)	6.1 (0.8)	5.6 (0.9)	0.002	0.79
Poor self-rated health, %	23.4 (1.3)	17.8 (1.3)	17.7 (1.4)	15.6 (1.3)	18.4 (1.3)	0.003	0.002
Cognitive function memory score, mean	16.3 (0.1)	17.3 (0.1)	17.2 (0.1)	17.4 (0.1)	17.3 (0.1)	<0.0001	0.0002
High depression symptoms, %	13.8 (1.1)	8.6 (1.0)	9.9 (1.1)	12.1 (1.0)	10.5 (1.1)	0.34	0.04
No educational qualification, %	29.2 (1.4)	22.7 (1.4)	19.2 (1.5)	21.6 (1.3)	18.5 (1.4)	<0.0001	0.01
Lowest wealth quintile, %	27.4 (1.4)	18.6 (1.3)	17.6 (1.4)	17.8 (1.3)	18.7 (1.4)	<0.0001	<0.0001
Lives with a partner, %	68.9 (1.5)	75.3 (1.5)	74.3 (1.5)	73.8 (1.4)	77.9 (1.5)	0.0004	0.48
Current smoking, %	11.9 (1.1)	10.5 (1.1)	11.8 (1.1)	11.3 (1.1)	14.4 (1.1)	0.09	0.08
Sedentary/Low activity, %	32.1 (1.4)	22.4 (1.4)	21.5 (1.5)	21.2 (1.4)	21.5 (1.4)	<0.0001	0.0001
Daily alcohol intake, %	23.8 (1.5)	23.0 (1.5)	25.5 (1.5)	22.1 (1.4)	23.0 (1.5)	0.59	0.64
Self-reported hearing impairment %	18.1 (1.3)	18.8 (1.3)	17.4 (1.3)	15.4 (1.2)	16.3 (1.3)	0.08	0.95

Abbreviations: IGF-1, insulin-like growth factor 1; BMI, body mass index. ^a^Values are means or percentages (standard error). ^b^IGF-1 (nmol/L) range in each quintile: 3–12.5; 13–15; 15.5–17.5; 18–20.5; 21–52 (men) and 4–11; 11.5–13.5; 14–15.5; 16–19; 19.5–43 (women).

**Figure 2:**
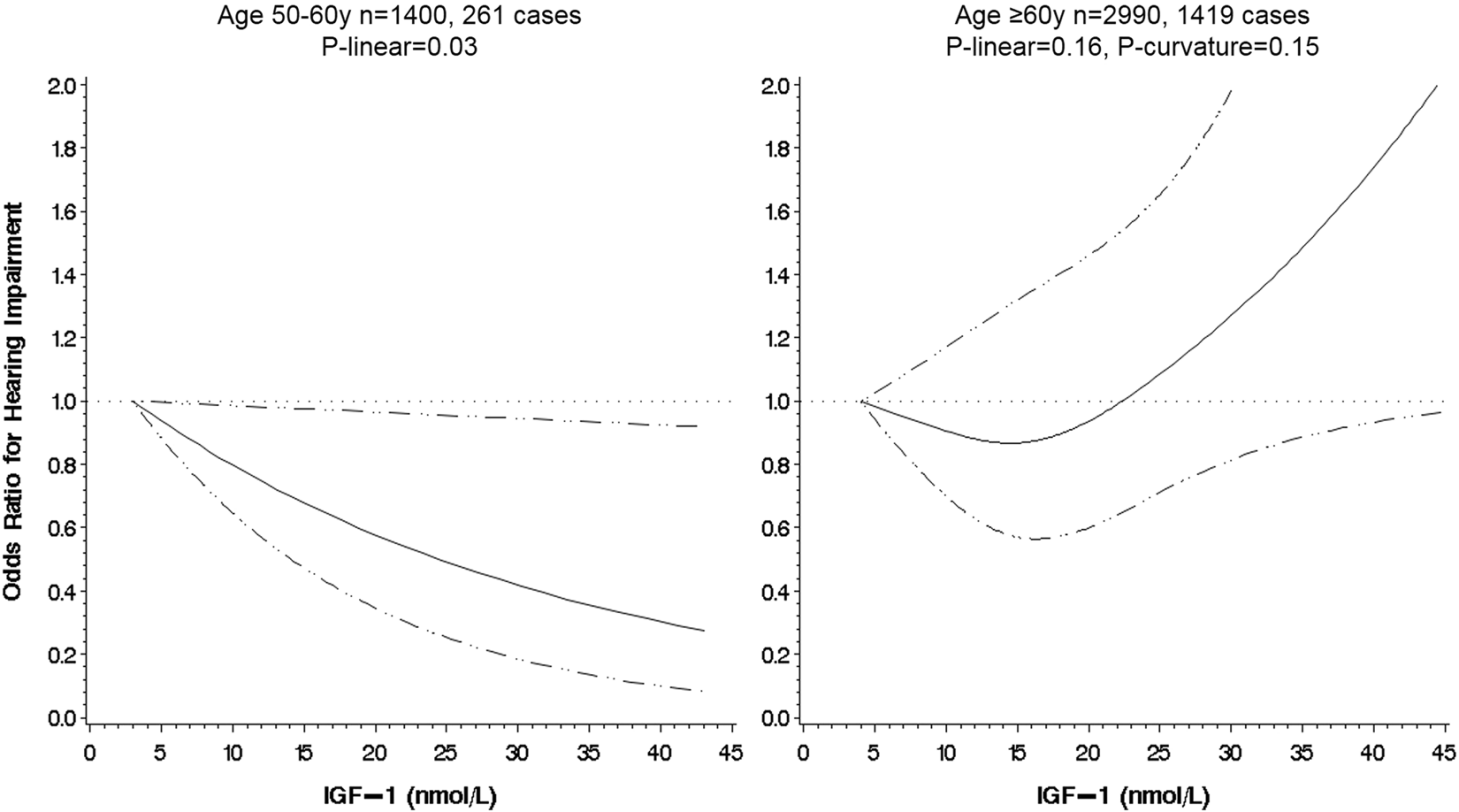
Serum Insulin-like Growth Factor-1 and odds of hearing impairment, by age group, the English Longitudinal Study of Ageing. Association estimated by logistic regression based on restricted cubic splines, the reference value is the minimum. Dashed lines indicate the 95% CIs. The model was adjusted for age, sex, height, smoking status, BMI, cognitive function (baseline and change), educational level, physical activity, self-rated poor health (baseline and change), and self-rated hearing acuity at baseline.

In the 2014 survey, of 4390 participants there were 1680 cases of objectively measured hearing impairment (prevalence 38.3%). The association between IGF-1 levels and subsequent hearing impairment are shown in Table 2. In the full analytical sample, there was no association between IGF-1 and hearing impairment. A strong interaction was found with baseline age (p for interaction = 0.03), but not with sex (p = 0.60), so we present the results for men and women combined and separately for those aged 50- < 60 and ≥60 years old (Fig. 2). In the younger group, there was a statistically significant inverse linear association that remained after multiple adjustment whereby higher levels of IGF-1 were linked to lower odds of future hearing loss: OR per 5nmol/L increase in IGF-1; 95% CI: 0.86; 0.73, 1.00, p = 0.03. In the older group, the association was non-linear (in Model 0, p-value for curvature = 0.02), however, none of the associations were statistically significant at conventional levels.

**Table 2:** Odds ratios (95% confidence interval) for hearing impairment in 2014 in relation to mean values of IGF-1 measured in 2008 and 2012: the English Longitudinal Study of Ageing (n = 4390).

Model	Sex-specific IGF-1 (nmol/L) quintiles^a^					OR continuous^b^	P-value linear^c^	P-value curvature^d^
	1	2	3	4	5			
	**Total sample n = 4390, 1680 cases**							
Model 0^e^	1 (ref)	0.90 (0.73; 1.11)	0.95 (0.77; 1.18)	0.87 (0.70; 1.07)	1.04 (0.84; 1.28)	1.00 (0.93; 1.07)	0.97	0.05
Model 1^f^	1 (ref)	1.00 (0.80; 1.24)	1.05 (0.85; 1.32)	0.99 (0.80; 1.23)	1.14 (0.91; 1.41)	1.02 (0.95; 1.09)	0.53	0.45
Model 2^g^	1 (ref)	0.94 (0.75; 1.18)	1.04 (0.83; 1.31)	0.95 (0.76; 1.19)	1.11 (0.88; 1.40)	1.01 (0.94; 1.09)	0.79	0.29
	**Age < 60 years, n = 1400, 261 cases** ^**h**^							
Model 0^e^	1 (ref)	0.72 (0.48; 1.09)	0.89 (0.60; 1.34)	0.62 (0.40; 0.94)	0.61 (0.40; 0.94)	0.82 (0.71; 0.95)	0.007	0.96
Model 1^f^	1 (ref)	0.83 (0.54; 1.27)	1.05 (0.69; 1.60)	0.75 (0.48; 1.16)	0.72 (0.46; 1.14)	0.87 (0.75; 1.00)	0.05	0.40
Model 2^g^	1 (ref)	0.80 (0.52; 1.24)	1.03 (0.66; 1.59)	0.69 (0.44; 1.10)	0.71 (0.44; 1.13)	0.86 (0.73; 1.00)	0.03	0.67
	**Age ≥60 years, n = 2990, 1419 cases** ^**h**^							
Model 0^e^	1 (ref)	0.91 (0.72; 1.16)	1.02 (0.79; 1.31)	0.89 (0.69; 1.14)	1.20 (0.94; 1.54)	1.06 (0.98; 1.15)	0.14	0.02
Model 1^f^	1 (ref)	0.98 (0.76; 1.25)	1.11 (0.86; 1.44)	0.98 (0.76; 1.27)	1.28 (0.99; 1.65)	1.07 (0.99; 1.16)	0.07	0.18
Model 2^g^	1 (ref)	0.93 (0.72; 1.21)	1.10 (0.84; 1.45)	0.96 (0.73; 1.26)	1.24 (0.95; 1.62)	1.06 (0.98; 1.16)	0.16	0.15

^a^IGF-1 (nmol/L) range in each quintile in the total sample: 3–12.5; 13–15; 15.5–17.5; 18–21; 21.5–52 (men) and 4–11; 11.5–13.5; 14–15.5; 16–19; 19.5–43 (women). Range in the younger group: 3–13.5; 14–16; 16.5–18; 18.5–21; 21.5–34.5 (men) and 4.5–12; 12.5–14.5; 15–17; 17.5–20; 20.5–43 (women). Range in the older group: 4–12; 12.5–15; 15.5–17.5; 18–20.5; 21–52 (men) and 4–10.5; 11–13; 13.5–15; 15.5–18; 18.5–38 (women). ^b^Odds ratio (95% confidence interval) for the increase of 5 nmol/L of IGF-1 concentration when IGF-1 is included as a continuous variable in the model. ^c^p-value of the effect of IGF-1 included as continuous in the regression model. ^d^p-value derived comparing a nested model with a linear or linear and cubic spline terms. If the p-value is low (<0.05), this denotes evidence of a non-linear relation. ^e^Model 0: Adjusted for age (continuous) and sex. ^f^Model 1: Model 0 + height (continuous), smoking status (never, ex-smoker, current), body mass index (BMI, continuous kg/m^2^), cognitive function (continuous score) at baseline and change, educational level (low, medium, high), physical activity (categorical, 5 levels), self-rated poor health (binary) at baseline and change. ^g^Model 2: Model 1 + self-rated hearing acuity at baseline. ^h^P-value for interaction term between IGF-1 and age was 0.03 (Model 0), 0.05 (Model 1) and 0.10 (Model 2).

**Figure 3:**
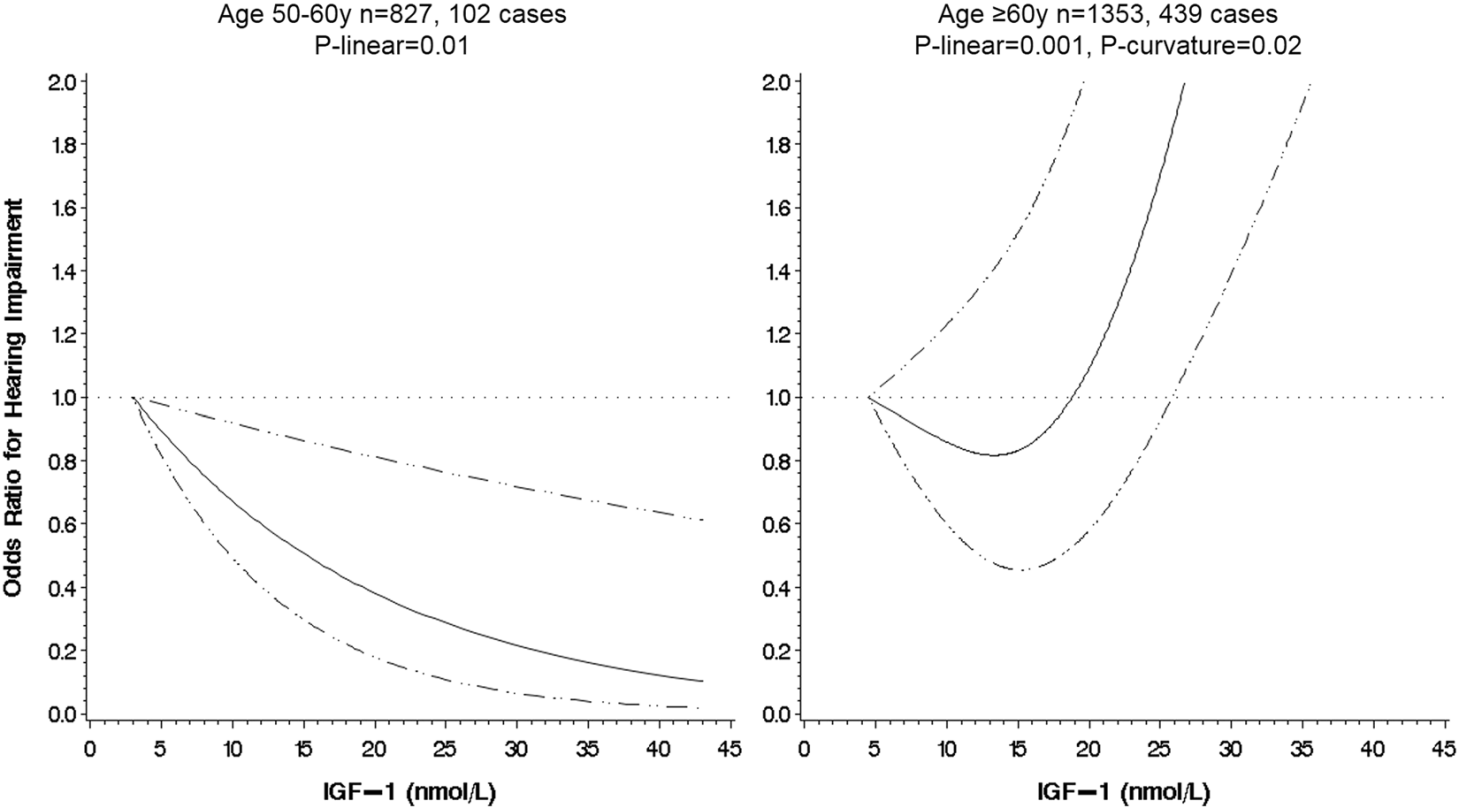
Serum Insulin-like Growth Factor-1 and odds of hearing impairment, by age group, after exclusion of self-reported mild to severe hearing impairment at baseline, the English Longitudinal Study of Ageing. Association estimated by logistic regression based on restricted cubic splines, the reference value is the minimum. Dashed lines indicate the 95% CIs. The model was adjusted for age, sex, height, smoking status, BMI, cognitive function (baseline and change), educational level, physical activity, self-rated poor health (baseline and change), and self-rated hearing acuity at baseline.

When we excluded the participants who self-reported poor, fair or good hearing at baseline to get closer to modelling the ‘incidence’ of hearing impairment among participants free of hearing impairment at baseline, there were 541 incident cases in 2176 participants. These participants were younger (mean age 62.9 y), more often women (60%) and displayed overall a more “favourable” health and behaviour profile. The results in these analyses also showed no significant association overall but a strong interaction with age was detected (p = 0.005). In the younger group (<60 y), the association was negative too and somewhat stronger than in the main analysis, with an OR of 0.38 (0.18; 0.81) when comparing the upper to the lower quintile of IGF-1. In the older group, evidence of a ‘J’-shape was clearer with a p-curvature = 0.02 in the fully adjusted model and increased odds of hearing impairment were found in the top IGF-1 quintile compared to the bottom (Table 3 and Fig. 3).

**Table 3:** Odds ratios (95% confidence interval) for hearing impairment in 2014 in relation to mean values of IGF-1 measured in 2008 and 2012 after exclusion of self-reported mild to severe hearing impairment at baseline: the English Longitudinal Study of Ageing (n = 2180).

Model	Sex-specific IGF-1 (nmol/L) quintiles^a^					OR continuous^b^	P-value linear ^c^	P-value curvature^d^
	1	2	3	4	5			
	**Total sample n = 2176, 541 cases**							
Model 0^e^	1 (ref)	0.82 (0.59; 1.13)	0.85 (0.62; 1.17)	0.95 (0.68; 1.33)	1.07 (0.77; 1.48)	1.05 (0.94; 1.17)	0.40	0.03
Model 1^f^	1 (ref)	0.93 (0.67; 1.30)	0.91 (0.66; 1.25)	1.11 (0.79; 1.57)	1.21 (0.86; 1.70)	1.09 (0.98; 1.21)	0.14	0.16
Model 2^g^	1 (ref)	0.92 (0.66; 1.28)	0.91 (0.66; 1.26)	1.08 (0.76; 1.53)	1.20 (0.85; 1.68)	1.08 (0.97; 1.21)	0.16	0.14
	**Age <60 years, n = 826, 102 cases** ^**h**^							
Model 0^e^	1 (ref)	0.73 (0.41; 1.32)	0.63 (0.34; 1.19)	0.61 (0.33; 1.13)	0.34 (0.16; 0.70)	0.72 (0.58; 0.89)	0.003	0.72
Model 1^f^	1 (ref)	0.77 (0.42; 1.42)	0.67 (0.35; 1.28)	0.71 (0.37; 1.35)	0.39 (0.18; 0.82)	0.75 (0.60; 0.94)	0.01	0.55
Model 2^g^	1 (ref)	0.76 (0.41; 1.39)	0.67 (0.35; 1.27)	0.70 (0.37; 1.33)	0.38 (0.18; 0.81)	0.75 (0.60; 0.94)	0.01	0.55
	**Age ≥60 years, n = 1350, 439 cases** ^**h**^							
Model 0^e^	1 (ref)	0.86 (0.59; 1.26)	0.97 (0.65; 1.43)	0.98 (0.67; 1.45)	1.56 (1.05; 2.30)	1.21 (1.06; 1.37)	0.004	0.01
Model 1^f^	1 (ref)	0.92 (0.62; 1.37)	1.02 (0.68; 1.53)	1.11 (0.75; 1.66)	1.72 (1.15; 2.59)	1.25 (1.10; 1.43)	0.001	0.02
Model 2^g^	1 (ref)	0.91 (0.61; 1.36)	1.03 (0.69; 1.55)	1.09 (0.73; 1.63)	1.71 (1.14; 2.57)	1.24 (1.09; 1.42)	0.001	0.02

^a^IGF-1 (nmol/L) range in each quintile in the total sample: 3–13; 13.5–15.5; 16–18; 18.5–20.5; 21–35 (men) and 4.5–11; 11.5–13.5; 14–16; 16.5–19; 19.5–43 (women). Range in the younger group: 3–13.5; 14–16; 16.5–18; 18.5–21; 21.5–34.5 (men) and 4.5–12; 12.5–14.5; 15–17; 17.5–20.5; 21–43 (women). Range in the older group: 6.5–12.5; 13–15; 15.5–17.5; 18–20.5; 21–52 (men) and 4.5–10.5; 11–13; 13.5–15; 15.5–18; 18.5–38 (women). ^b^Odds ratio (95% confidence interval) for the increase of 5 nmol/L of IGF-1 concentration when IGF-1 is included as a continuous variable in the model. ^c^p-value of the effect of IGF-1 included as continuous in the regression model. ^d^p-value derived comparing a nested model with a linear or linear and cubic spline terms. If the p-value is low (<0.05), this denotes evidence of a non-linear relation. ^e^Model 0: Adjusted for age (continuous) and sex. ^f^Model 1: Model 0 + height (continuous), smoking status (never, ex-smoker, current), body mass index (BMI, continuous kg/m^2^), cognitive function (continuous score) at baseline and change, educational level (low, medium, high), physical activity (categorical, 5 levels), self-rated poor health (binary) at baseline and change. ^g^Model 2: Model 1 + self-rated hearing acuity at baseline (excellent, very good). ^h^P-value for interaction term between IGF-1 and age was 0.005 (Model 0), 0.008 (Model 1) and 0.01 (Model 2).

In another set of sensitivity analyses (Supplemental Fig. 1), we included only those participants with two measures of IGF-1, resulting in a sample of 3085 (n = 1153 cases of hearing impairment). Again, a similar pattern of results was apparent to those seen in the main analysis but precision was lower as evidenced by wider confidence intervals. When we used only one IGF-1 measure (at baseline), results were also similar to the main analysis (Supplemental Fig. 2). Finally, when we modelled moderate to severe hearing impairment, there were 580 cases (15%), of which 530 (92%) occurred in participants aged >60y at baseline. There was no statistically significant interaction with age (p = 0.24) and the overall association followed a significant ‘J’-shaped curve in Model 0, which did not remain overall significant after multiple adjustment (Supplemental Fig. 3).

## Discussion

In this national cohort of older English adults, we found no association between circulating levels of IGF-1 and subsequent hearing impairment. However, the relationship appeared to differ according to age such that there was lower odds of hearing impairment observed at higher levels of IGF-1 among people aged below 60 years. In participants aged 60 and over, there was evidence of a ‘J’-shape, with higher odds of hearing impairment observed at higher levels of IGF-1.To our knowledge, this is the first study to assess the association between IGF-1 and hearing impairment in a population-based study, despite the use of this hormone as an alternative treatment for sudden hearing loss in clinical trials^37^. In participants with no hearing impairment at baseline, the effect of IGF-1 can be regarded as moderately high^38^ in young older adults.

Animal studies suggest a direct role of IGF-1 in the development of hearing function, as IGF-1 deficiency has been shown to cause a number of cellular alterations in the mouse cochlea, leading to deafness^16, 18^. In humans, IGF-1 deficiency is rare and is associated with poor growth rates, mental retardation and hearing loss^18^. In turn, it has been suggested that IGF-1 is required for the correct development and maintenance of hearing and that IGF-1-based therapies could contribute to prevent age-related hearing loss^16^, despite the absence of evidence in population-based studies. In normal aging mice, circulating IGF-1 levels decrease with age, concomitantly with significant age-related hearing loss^16^. In humans, observational studies have shown a slow decline in IGF-1 circulating levels, happening in later older age^39^. This was confirmed in our study with a decreasing linear trend of age across IGF-1 quintiles. However, no significant higher levels of hearing impairment was observed at lower levels of IGF-1 among respondents aged 60 and over. IGF-1 plays a complex role in ageing processes, including cognitive function decline and chronic disease development. For instance, it has been suggested that IGF-1 can have some neuroprotective effects but can also accelerate neurodegeneration^40^. Similarly, both very low and very high levels of IGF-1 have been associated with increased risk of mortality from cardiovascular disease or cancer^15^. Furthermore, mutations in the IGF-1 pathway genes – inducing reduced IGF-1 signalling - have been associated with extended longevity in some studies^41^. With conflicting potential mechanistic roles, IGF-1 has not been shown to be a good predictor of age-related disease outcomes such as cardiovascular disease, cancer or dementia.

Age-related hearing loss is a progressive disorder with people predominantly first affected after age 60 and is defined as hearing impairment mostly due to ageing of the cochlea^1^. Conversely, hearing impairment happening at middle age can be the result of many different factors including exposure to noise, ototoxic agents, otological disorders and genetic susceptibility^42^. IGF-1 has been used in clinical trials as an alternative cure to corticosteroids for sudden sensorineural hearing loss in middle-aged adults^37^, suggesting a protective effect that may act through prevention of hair cell death after exposure to conditions that cause inner ear pathology. A potential interpretation of the observed differential effect of IGF-1 according to age is that the protective role of IGF-1 may not apply as much in presbycusis, where vascular damage may be a stronger risk factor. Indeed, age-related hearing loss has been shown to be aggravated by the presence of cardiovascular risk factors and higher pro-inflammatory states^43^. A complex role of IGF-1 and the IGF-1 axis has been suggested in atherosclerosis, as higher levels of IGF-1 can contribute to atherosclerotic plaque development in restenosis, while low levels of IGF-1 contributes to plaque destabilization^41, 44^, and its action is modulated by many other physiological parameters such as presence of insulin resistance or hypercholesterolemia. Similarly, both anti- and pro-inflammatory effects of IGF-1 have been described^41^. These complex effects of IGF-1 on vascular function may partly explain the ‘J’-shaped observed in relation to hearing impairment in the older group of our study.

### Study strengths and limitations

The main strength of this study is that it is a large, nationally representative sample of older people living in England with good follow-up. IGF-1 levels were measured at two separate occasions, and taking the average of these two measurements is a way to limit bias in the relationship with hearing impairment^30^. The outcome was objectively measured using a validated screening device also used in the Health Survey for England^45^. A major limitation is that it is an observational study so limiting causal insights. Despite carefully accounting for potential confounders, measurement error in their assessment, particularly in the self-reported covariates (e.g. smoking, self-rated health), may have led to residual confounding and biased the associations. Moreover, we did not have information on noise exposure which would have been an important factor to take into account. We also used an average of two measured of IGF-1 taken 4 years apart or only one measurement if only one was available, in order to improve accuracy and increase sample size. This may have attenuated the results but sensitivity analysis including only participants with both measurements led to similar conclusions but not statistically significant associations, and similarly when we only used IGF-1 measurement in 2008. We also had to exclude an important part of the sample who had missing data (26% of participants with complete baseline data did not have a follow-up measurement) and, compared to those included, excluded participants were older, less educated, engaging in less healthy behaviours (smoking, sedentary) and more likely to develop hearing impairment, hence also potentially biasing the observed estimates. However, even if those included participants are different from the general population, as long as there is enough variability in the measurements, generalisability of the associations should not be entailed. The point has recently been made in the UK Biobank study which has a response rate of only 5.5%^46^. Some studies have compared exposure-disease associations according to survey participation status, showing similar magnitudes. For example in the UK Health and Lifestyle Surveys, 29% of participants did not participate in resurvey 7 years later, but the associations between baseline cardiovascular risk factors such as hypertension and cardiovascular mortality did not differ by participation status^47^. We also acknowledge that this study does not strictly assess incidence of hearing impairment as hearing was tested objectively only at follow-up, while we used questionnaire data to assess self-rated hearing at baseline. Finally, we measured total IGF-1 levels, which are considered a crude estimate of the biologically active hormone, whereas free IGF-1, which represents less than 1% of total IGF-1, is believed to be the most active form and to have greater physiological and clinical relevance than total IGF-1^41^. However, results from animal studies showing a role in the development and maintenance of hearing function also measured total IGF-1^16, 17^, and total IGF-1 has been used as a biomarker in most epidemiological studies in relation to chronic disease^15^.

### Conclusions and implications

In people at the lower end of the older-aged spectrum (aged 50 to 60 years), higher levels of IGF-1 were associated with lower odds of hearing impairment and most particularly in participants free of hearing impairment at baseline, where a moderately high effect size was found. This is the first population-based study to show a linear relationship between IGF-1 and hearing impairment. We found significant 14% decrease odds for a relatively small increase in IGF-1 (5nmol/L). The ‘J’-shaped relationship observed at older ages may partly reflect the association of IGF-1 with vascular health. With currently no existing cure for hearing loss, identification of relevant risk factors remains a priority. More observational studies are needed to confirm our results before considering the possibility of testing low-dose supplementation in IGF-1 for long-term prevention of hearing impairment.

## Electronic supplementary material

Supplemental Figures 1-3
