# Supplementary material for: Insulin-like Growth Factor 1 in relation to future hearing impairment: findings from the English Longitudinal Study of Ageing
Source: Sci Rep. 2017 Jun 23;7:4212. doi: 10.1038/s41598-017-04526-7 (PMC5482884; doi:10.1038/s41598-017-04526-7)
Supplement: Supplementary file 1 — Supplemental Figures 1-3 [file 41598_2017_4526_MOESM1_ESM.doc]

**Supplemental Figure 1. Serum Insulin-like Growth Factor-1 and odds of hearing impairment in participants with two measurements on IGF-1 (2008 and 2012), by age group**

**
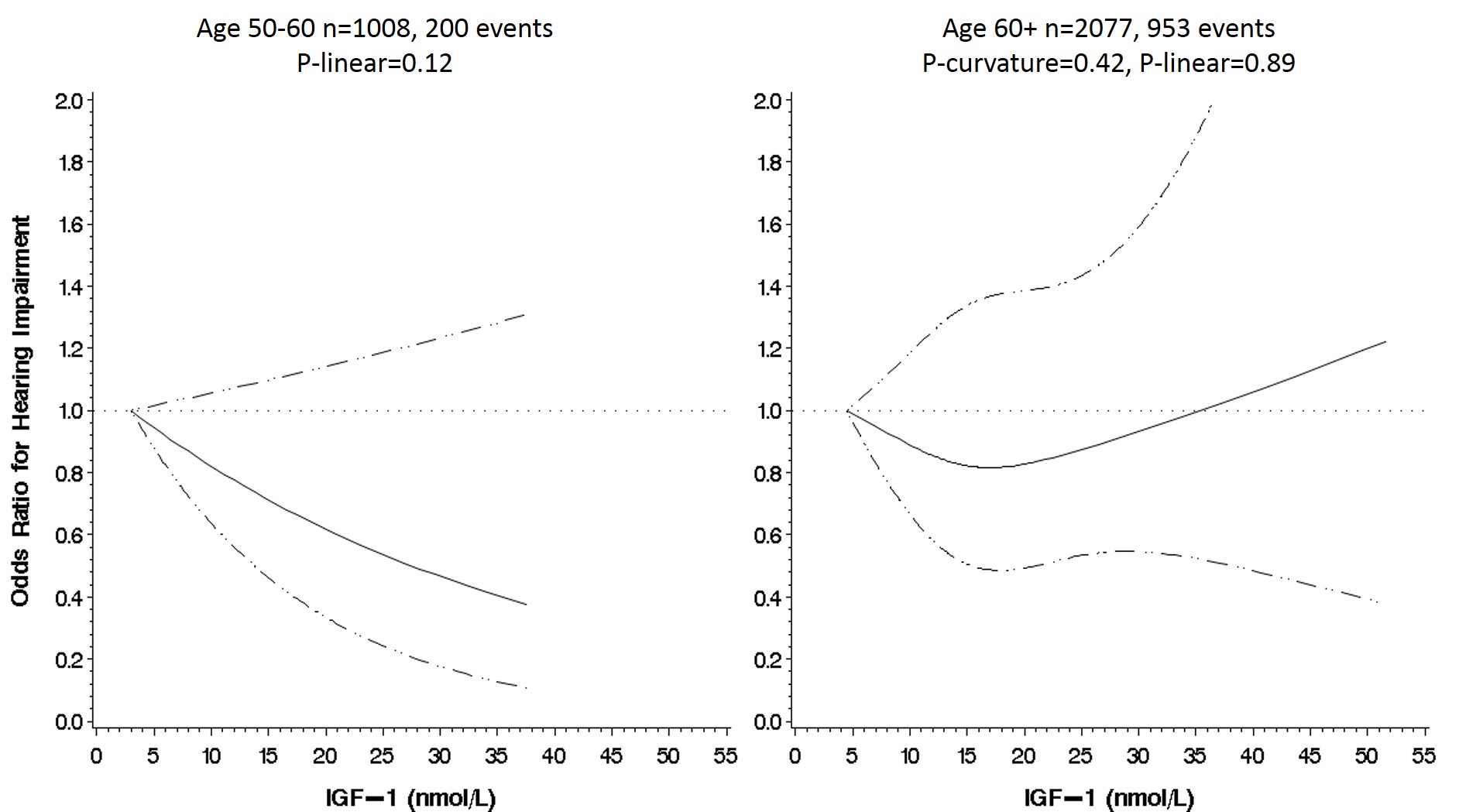
**

Association estimated by logistic regression based on restricted cubic splines, the reference value is the minimum. Dashed lines indicate the 95% CIs. The model was adjusted for age, sex, height, smoking status, BMI, cognitive function baseline and change, educational level, physical activity, self-rated poor health baseline and change, and self-rated hearing acuity at baseline.

**Supplemental Figure 2. Serum Insulin-like Growth Factor-1 and odds of hearing impairment where IGF-1 was assessed only once at baseline (2008), by age group**


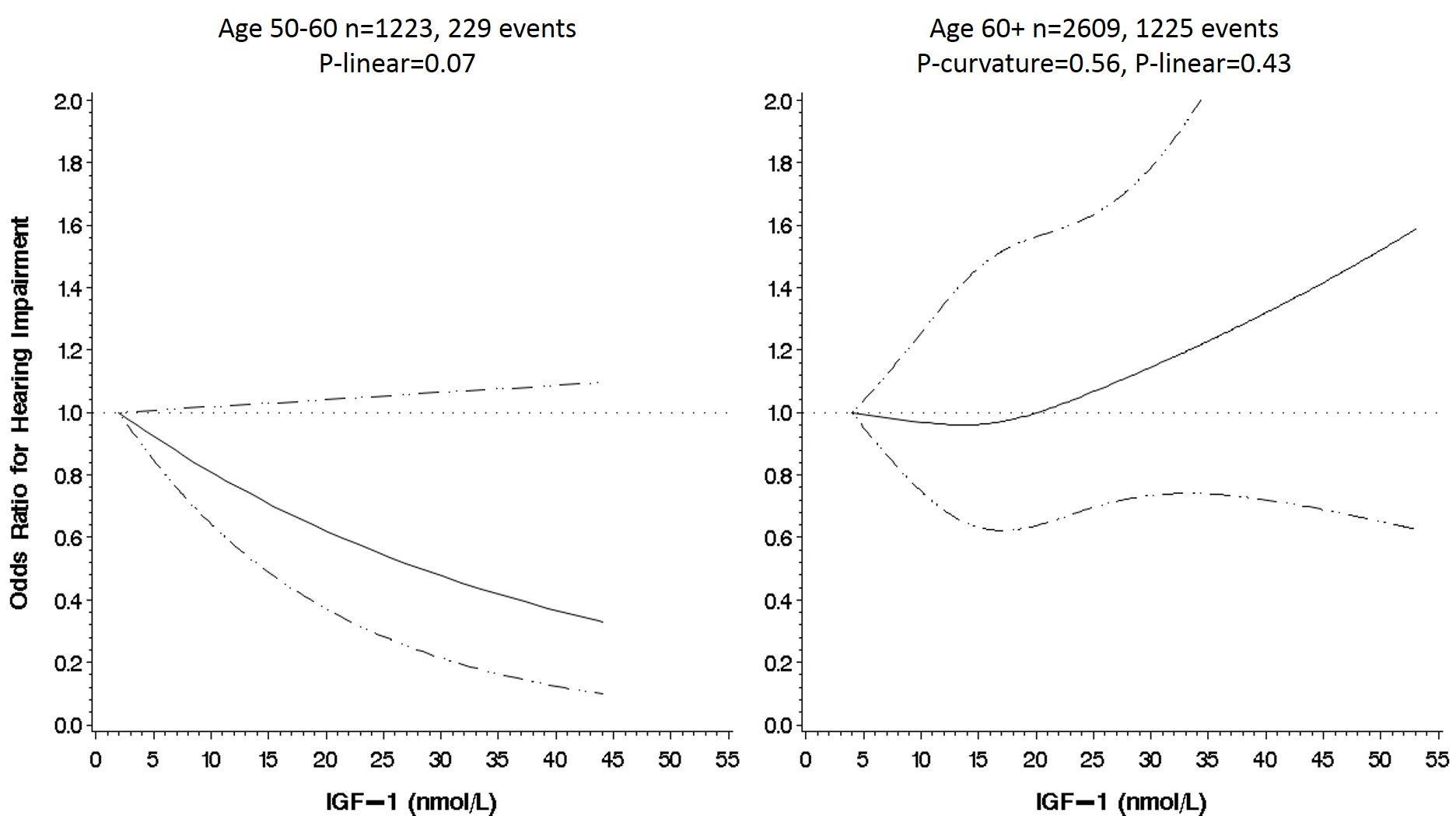
Association estimated by logistic regression based on restricted cubic splines, the reference value is the minimum. Dashed lines indicate the 95% CIs. The model was adjusted for age, sex, height, smoking status, BMI, cognitive function baseline and change, educational level, physical activity, self-rated poor health baseline and change, and self-rated hearing acuity at baseline.

**Supplemental Figure 3.** **Serum Insulin-like Growth Factor-1 and odds of moderate to severe hearing impairment**


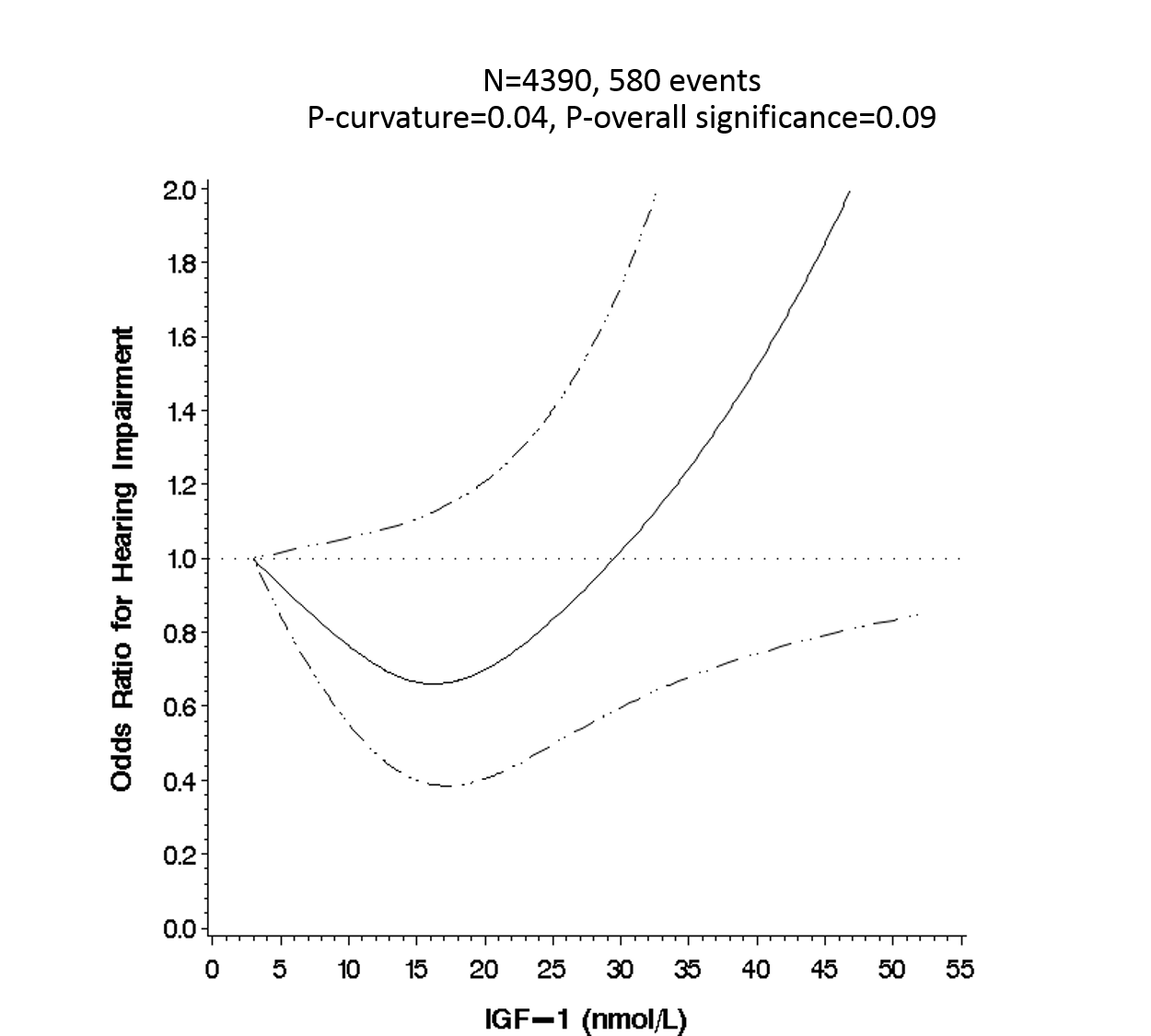


Association estimated by logistic regression based on restricted cubic splines, the reference value is the minimum. Dashed lines indicate the 95% CIs. The model was adjusted for age, sex, height, smoking status, BMI, cognitive function baseline and change, educational level, physical activity, self-rated poor health baseline and change, and self-rated hearing acuity at baseline.
